# Analysis of rescue strategies for acute thrombosis during STA-MCA bypass surgery and the literature review

**DOI:** 10.1007/s00701-024-06194-9

**Published:** 2024-07-23

**Authors:** Haitao Xu, Haojin Ni, Chenhui Zhou, Xinwen Wang, Jie Wei, Tiansheng Qian, Zifeng Dai, Wenting Lan, Xin Wu, Jiacheng Yu, Xianru Li, Xiang Gao, Bin Xu, Jinghui Lin

**Affiliations:** 1 The First Affiliated Hospital, Health Science Center, Ningbo University, Ningbo, China; 2 Department of Neurosurgery, Ningbo Key Laboratory of Nervous System and Brain Function, The First Affiliated Hospital of Ningbo University, Ningbo, Zhejiang 315010 China; 3 Department of Neurology, The First Affiliated Hospital of Ningbo University, Ningbo, China; 4 Department of Radiology, The First Affiliated Hospital of Ningbo University, Ningbo, Zhejiang China; 5 Department of Neurosurgery, Huashan Hospital, Fudan University, Shanghai, China

**Keywords:** Moyamoya disease, Bypass, Acute thrombosis, Tirofiban

## Abstract

**Background and objectives:**

STA-MCA bypass surgery is mainly used for Moyamoya disease, giant intracranial aneurysms, and resection of intracranial tumors requiring sacrifice of blood vessels. The intraoperative patency of the reconstructive vessels is critical to the efficacy of the procedure. This study aimed to evaluate the efficacy of intra-arterially infused tirofiban for the treatment of acute thrombosis during STA-MCA bypass surgery and countermeasures for acute thrombosis.

**Methods:**

This study involved 209 patients (272 hemispheres) who underwent STA-MCA surgery between November 2020 and December 2023. Intraoperative acute thrombosis occurred in eight patients (3.83%,8 hemispheres). We retrospectively reviewed the clinical and imaging data, surgical procedure, and follow-up outcomes of eight patients. We implemented the different thrombolytic methods to evaluate the optimal thrombosis management during the bypass surgery. After three months, we assessed neurological functions using the modified Rankin Scale (mRS) and conducted a literature review using PubMed.

**Results:**

Eight patients (four male patients and four female patients) developed acute thrombosis during the bypass surgery. Of the eight patients, two underwent re-anastomosis after thrombus removal, three received local injections of tirofiban into the anastomosis or the branches of the superficial temporal artery, and three underwent superselective intra-arterial tirofiban infusion using a microcatheter. Thrombosis were resolved, and arteries were recanalized in all patients. The mRS score was 0 in all patients. No major ischemic or hemorrhagic complications occurred.

**Conclusion:**

Our treatment methods were efficacious in the management of acute thrombosis. Intra-arterial tirofiban administration seems to be a simple and effective treatment option for acute thrombosis during STA-MCA bypass surgery.

## Introduction

STA-MCA bypass surgery is mainly used for Moyamoya disease, giant intracranial aneurysms, and resection of intracranial tumors requiring sacrifice of blood vessels [14, 16, 19]. Previous studies reported that direct bypass is more efficacious. Greater angiographic collateralization, superiority in restoring cerebrovascular reserve capacity (CVRC), increased symptomatic improvement, and longer stroke-free survival are the primary benefits [11, 22]. In terms of the direct revascularization procedure, recent reports have shown that the incidence of acute thrombosis at the anastomosis site ranges from 3.85% to 9.30% [8, 12, 15]. Similar to intracranial arterial thrombosis, an anastomotic thrombus, if not promptly resolved, may result in direct vascular reconstruction failure. Thrombus expansion could provoke occlusion of the recipient artery, triggering related ischemic complications such as stroke or neurological functional impairment. The success of bypass surgery depends on the degree of anastomotic patency [13]. We implemented three distinctive approaches for patients who developed acute thrombosis during STA-MCA procedures. These encompassed re-anastomosis post-thrombus extraction, tirofiban injections through the anastomosis or the branches of the STA, and superselective intra-arterial tirofiban infusion using microcatheter.

## Methods

### Patients

This study involved 209 patients (272 hemispheres) who underwent STA-MCA surgery between November 2020 and December 2023. Intraoperative acute thrombosis occurred in eight patients (3.83%;8 hemispheres;6 MMD patients, 1middle cerebral artery occlusion,1internal carotid artery occlusion). Of the eight patients, two underwent re-anastomosis after thrombus removal, three received local tirofiban injection into the anastomosis or the branches of the STA, and three underwent superselective intra-arterial tirofiban infusion using microcatheter. Aspirin therapy commenced one week preoperatively, and postoperatively, administration of aspirin began on the second day and continued uninterrupted. Three months later, we assessed neurological function using the modified Rankin Scale (mRS). Good clinical outcome at 3 months was defined as mRS 0–2. All patients provided consent for the publication of their images. The Institutional Review Board of our hospital approved this study.

### Assessment of acute thrombosis

The STA-MCA bypass surgery was performed by an experienced neurosurgeon. We selected donor vessels with a diameter exceeding 1mm. All eight patients in the study used the parietal STA branch. After isolating the terminal branches of the superficial temporal artery, a temporary aneurysm clip is applied to the proximal STA donor, while the distal STA is ligated and divided to the appropriate length. We utilized a bipolar cutting technique to dissect the adherent soft tissues from the donor artery wall. The vessel is then flushed with heparinized saline. An arteriotomy is executed by excising an elliptical portion from the superior wall of the recipient vessel. The anastomosis was performed end-to-side, secured with approximately 8–12 stitches of 10–0 prolene thread. Interrupted suture technique was used. Firstly, the front wall of the anastomosis was meticulously sutured. When suturing the back wall, initial sutures were passed through the vessel and secured using the interrupted suturing technique. The last few sutures were passed through the vessel but temporarily left unknotted. Following this, the anastomosis site was flushed with heparin, and a thorough inspection was conducted through the untied portion of the anastomosis to ensure no thrombus formation, opposite wall suturing, or other technical errors. Finally, the remaining sutures were carefully knotted. Mean time to recipient vessel blockade was 18min28s. The patency was verified post-bypass through indocyanine green(ICG) [1, 17]. When occlusion occurred at the anastomotic site, we conducted an initial assessment outside the vessel to detect any thrombus formation. This involved assessing for muscle tension in the temporal region, examining the folding of the STA, and using local massages or gentle artery wall compression with micro-forceps alongside local papaverine injections. Despite these measures, patients with persistent occlusion were suspected to have developed an anastomotic thrombus, which was subsequently confirmed through suture removal or digital subtraction angiography(DSA) imaging.

### Countermeasures in acute thrombosis

The initial approach involves the direct extraction of thrombosis. Initially, the incision of the donor artery opens the anastomosis, allowing the thrombosis to be retrieved. After complete thrombosis removal, refining the rough tissue from the opening of the recipient artery and the temporal superficial artery creates a pristine surface. After that, a complete re-anastomosis was performed from start to finish.

After the removal of 1–2 stitches at the anastomosis site, the second approach involves injecting 50ug tirofiban into partially opened anastomoses and the STA branch. Upon observation of thrombus dissolution and assessment of anastomotic patency using ICG, followed by thorough irrigation and subsequent reanastomosis.

The third approach stems from our previous experience. In our previous studies, we employed the second method for addressing anastomotic thrombosis, achieving successful thrombus removal. However, considering the potential risk of damaging the vascular endothelium by removing certain anastomotic sutures, we endeavored to preserve these sutures. After induction of general anesthesia, the patient was placed in a supine position with the head inclined at a 60-degree angle to the contralateral side. The groin was sterilized and draped, facilitating access to the right femoral artery using the Seldinger technique, followed by insertion of a 5 F sheath. Subsequently, a 5F curved catheter was selected and advanced to the beginning of parietal branch of superficial temporal artery on the affected side. In the event of intraoperative acute thrombosis, the microcatheter tip is adjusted to position within 1-2cm proximal to the thrombus. Following this adjustment, 1ml of 50ug/ml tirofiban is infused into the artery via the microcatheter, initiating a 10-min interval before angiography. Should the angiogram unveil persistent occlusion at the anastomotic site, an additional 1ml of 50ug/ml tirofiban is administered through the microcatheter, and a repeat angiography is conducted. Upon confirmation of vessel patency, further validation of anastomotic patency is performed using ICG.

**Fig. 1:**
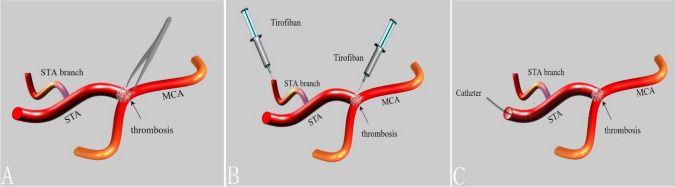
A simple illustration of the three methods. **A** re-anastomosis after thrombus removal. **B** local injection of tirofiban into the anastomosis or the branches of the STA. **C**. superselective intra-arterial tirofiban infusion via a microcathe

A simple illustration of the three methods is shown in Fig. 1. All three methods involved a reassessment of anastomotic patency post-thrombus removal using ICG.

### Tirofiban Preparation

We used tirofiban hydrochloride sodium Injection, available in a 100 mL formulation comprising 5 mg of tirofiban hydrochloride and 0.9 g of sodium chloride. The solution is administered slowly and continuously through manual injection at a rate of 1ml/min (0.05mg/min tirofiban) [3]. A couple of 1-mL syringes were used for this infusion. Approximately 1 mL of the injection was administered per instance. The dose of tirofiban injected into the artery was 0.15–0.2 mg for 3–4 injections.

## Results

### Clinical characteristics

Table 1 shows the clinical characteristics of the eight patients. We included four male patients and four female patients in our series, aged 23–66 years. The initial symptoms in our patients included hemorrhagic stroke (n = 5) and ischemic stroke (n = 3).

**Table 1:** Patients clinical characteristics

Case no	Sex/Age	Symptoms	Operated side	Suture	Anastomosis patency	Method	mRs	3–6 months patency
1	Male/40	Hemorrhagic stroke	Left	interrupted sutures	Yes	3	0	Yes
2	Female/66	Ischemic stroke	Right	interrupted sutures	Yes	1	0	Yes
3	Female/23	Hemorrhagic stroke	Left	interrupted sutures	Yes	2	0	Yes
4	Female/50	Hemorrhagic stroke	Right	interrupted sutures	Yes	3	0	Yes
5	Male/59	Ischemic stroke	Left	interrupted sutures	Yes	3	0	No
6	Female/36	Hemorrhagic stroke	Left	interrupted sutures	Yes	2	0	No
7	Male/47	Hemorrhagic stroke	Left	interrupted sutures	Yes	1	0	Yes
8	Male/60	Ischemic stroke	Right	interrupted sutures	Yes	2	0	Yes

Method1:re-anastomosis after thrombus removal

Method2:local injection of tirofiban into the anastomosis or the branches of the STA

Method3:superselective intra-arterial tirofiban infusion via a microcatheter

### Procedure and outcome of three cases of salvage occluded bypasses

#### Case1

**Fig. 2:**
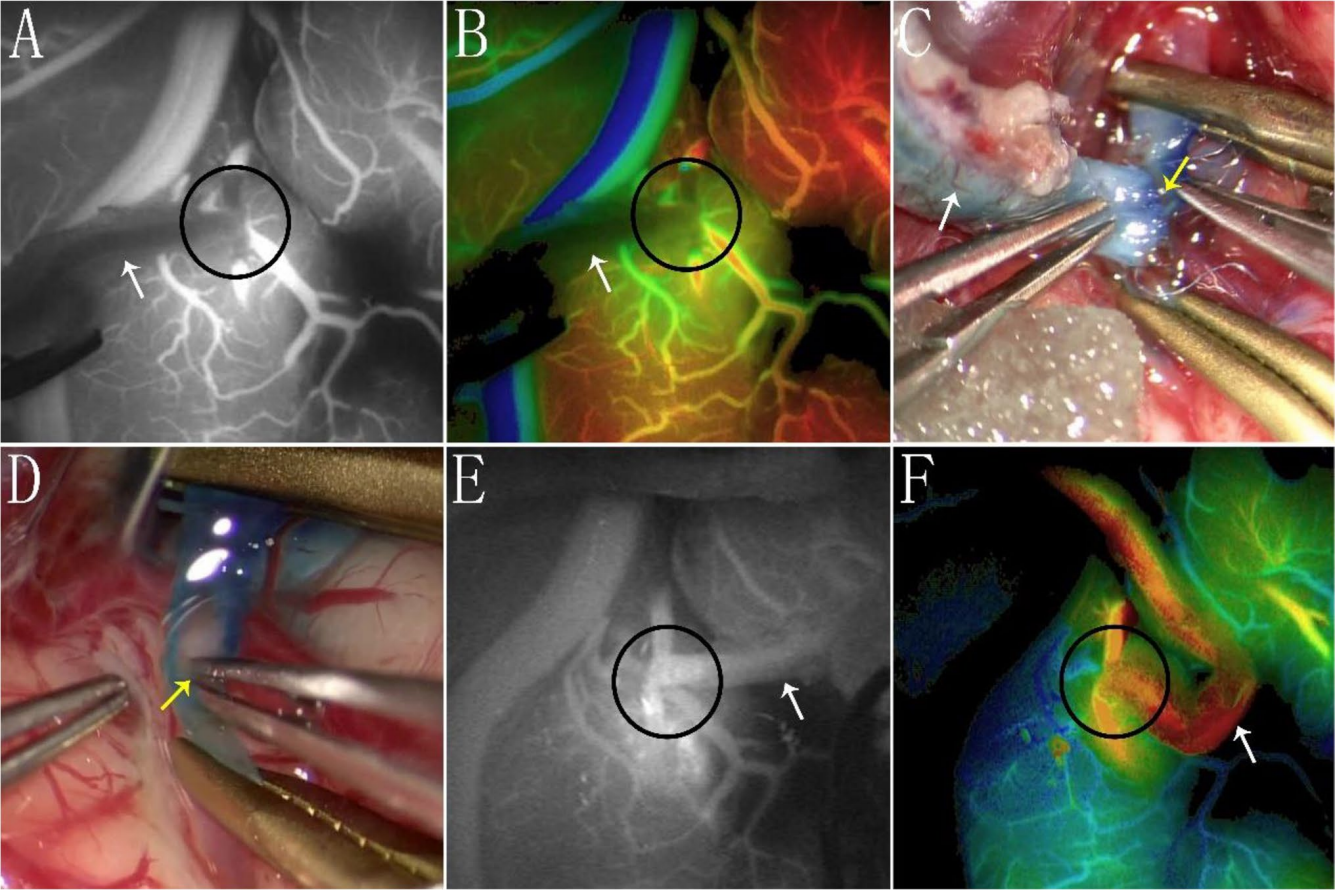
A case of thrombus extraction through incision. **A** ICG shows anastomotic occlusion. **B** Corresponding Flow 800 image to Image A. **C** Unpacking two sutures at the anastomosis revealed the presence of a thrombus. **D** The process of retrieving the thrombus. **E** ICG post-secondary anastomosis indicates patent anastomosis. **F** Flow 800 image post-secondary anastomosis. (black circle represents the anastomosis, white arrow denotes the STA, and the yellow arrow indicates the thrombus)

A 47-year-old man, diagnosed with Moyamoya disease, presented with intracranial hemorrhage as the primary manifestation. The initial right-sided STA-MCA bypass surgery proceeded seamlessly, yielding a favorable recovery for the patient. However, during the subsequent left-sided STA-MCA bypass surgery, an acute thrombotic complication manifested at the anastomotic site. Employing the first approach to address the acute thrombus. ICG and FLOW800 imaging validated the restoration of patency at the anastomosis (Fig. 2). No further complications were noted, and there were no associated adverse events observed.

#### Case2

**Fig. 3:**
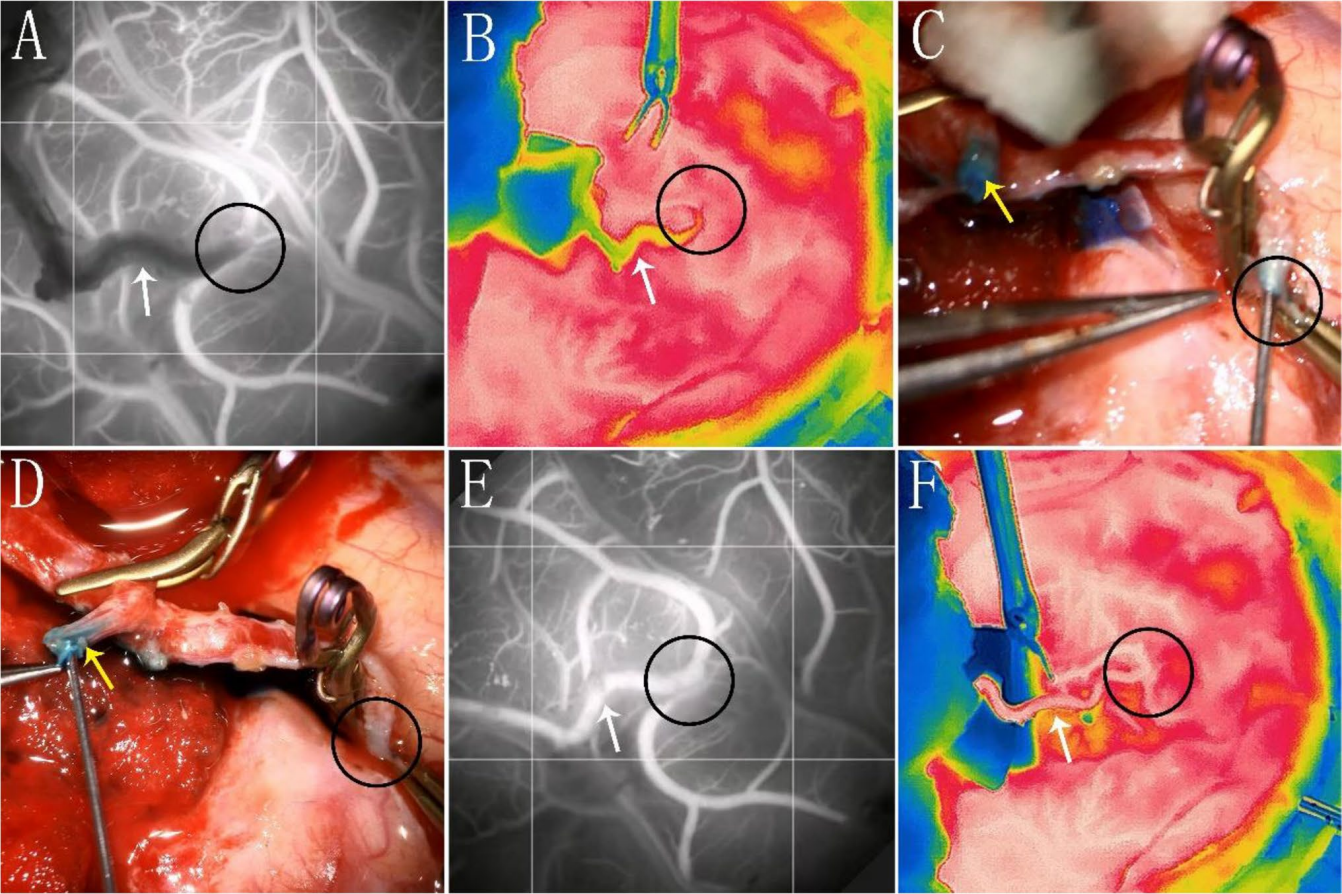
One case treated by injecting tirofiban through the branches of the STA or at the anastomosis site to manage thrombosis. **A** Intraoperative ICG revealed occlusion at the anastomosis site. **B** Corresponding infrared thermography image to A. **C** Injection of tirofiban at the anastomosis site. **D** Procedure of tirofiban injection into branches of the STA. **E** Post-reanastomosis, ICG exhibited restored patency at the anastomosis site. **F** Corresponding infrared thermography image to E. (Where the white arrow represents the STA, the yellow arrow depicts its branches, and the black circle denotes the anastomosis.)

A 36-year-old woman, admitted for intracranial hemorrhage stemming from Moyamoya disease, underwent left-sided STA-MCA bypass surgery. Following with initial vascular reconstruction, ICG imaging showed occlusion at the anastomotic site. Upon removing two sutures, thrombus formation was noted. We applied the second approach to address the thrombus, and ICG imaging and infrared thermography affirmed the patency of anastomosis (Fig. 3).

#### Case3

**Fig. 4:**
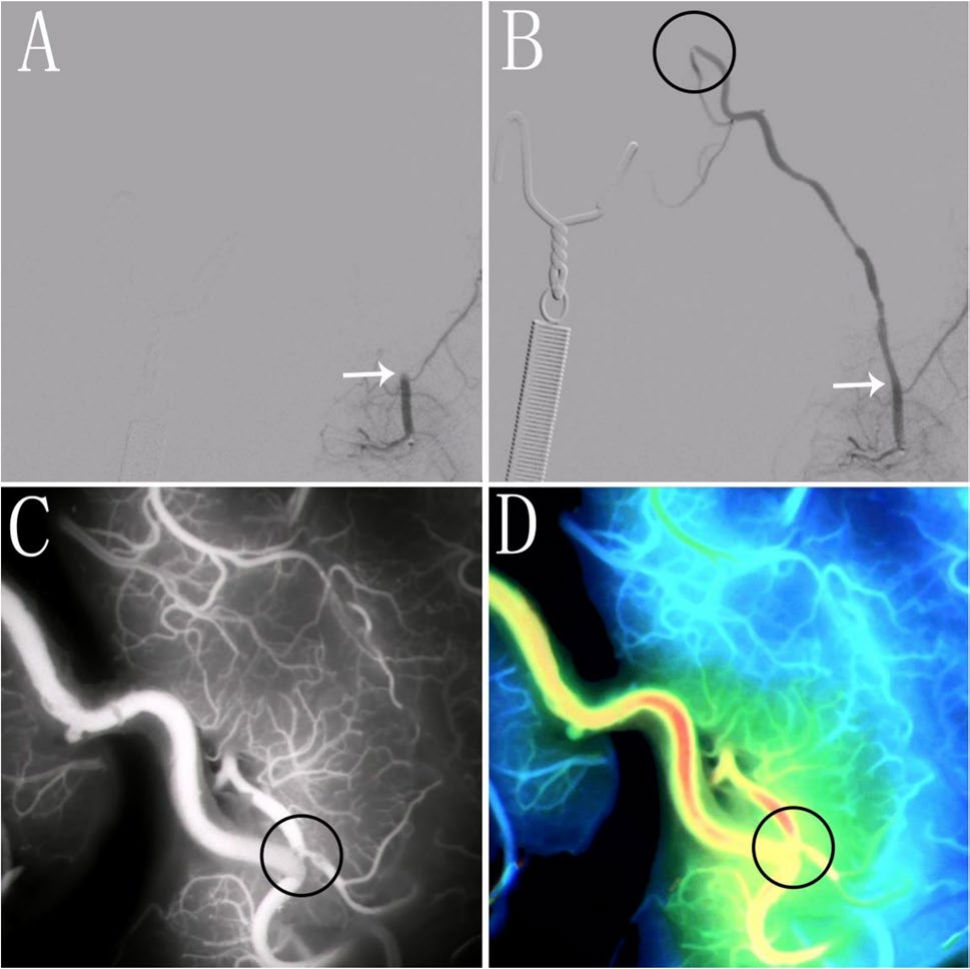
A case underwent superselective intra-arterial tirofiban infusion via a microcatheter. **A** Intraoperative DSA imaging post-reconstruction indicates the STA; however, blood flowfails to reach the anastomosis, suggesting impaired anastomotic patency. **B** DSA imaging post-tirofiban injection through the catheter shows blood flow at theanastomosis; the arrow signifies the location of blood flow during anastomotic closure. **C** Following confirmation of anastomotic patency via DSA, indocyanine green angiographywas employed once again, demonstrating restored patency at the anastomosis. **D** Image C corresponds to the FLOW800 image. (The black circle represents the anastomosis.)

A 59-year-old man was hospitalized following a sudden syncope episode. The etiology stemmed from a typical occlusion of the left internal carotid artery. The surgical plan included left hemisphere STA-MCA bypass surgery, during which an acute thrombus formed at the anastomosis. Employing the third approach to manage the thrombus, the patient received 0.15mg of tirofiban treatment, effectively resolving the thrombus at the anastomosis. Intraoperative ICG imaging and DSA confirmed patency at the anastomosis (Fig. 4). The patient recovered well, experiencing no associated complications.

Of the eight patients, two underwent re-anastomosis after thrombus removal, three received tirofiban injection through the anastomosis or STA branches, and three underwent superselective intra-arterial tirofiban infusion via a microcatheter. Thromboses were resolved, and arteries were recanalized in all patients. No hemorrhagic complications related to intra-arterial tirofiban infusion occurred.

### Literature review

Table 2 shows recent treatments for acute thrombosis encountered during bypass surgery. However, researchers have seldom explored the application of tirofiban in STA-MCA bypass surgery. Our study was the first to administer tirofiban for acute thrombosis during STA-MCA procedures.

**Table 2:** Strategies for managing acute thrombosis during EC-IC bypass surgery

Series (ref. no.)	Countermeasure in acute thrombosis
Kimura T et al., 2018[12]	1.re-anastomosis after thrombus removal
Takeshi Mikami et al., 2019[15]	1.local massage, local administration of papaverine hydrochloride and intravenous administration of heparin 2.the local tight binding should be untied and resutured
Winkler EA et al., 2021[23]	local in situ fibrinolysis with r-TPA
Jin Woo Bae et al., 2021[2]	heparin solution
Buchanan IA, et al., 2021[4]	abciximab
Takahashi JC et al., 2022[20]	1.Systemic heparinization.、Nasogastric administration of aspirin 2.re-anastomosis after thrombus removal
Current research	1.re-anastomosis after thrombus removal 2.local injection of tirofiban into the anastomosis or the branches of the STA 3.superselective intra-arterial tirofiban infusion via a microcatheter

## Discussion

Acute thrombus formation is a complication of direct revascularization surgery [15]. Establishment and maintenance of bypass patency are essential for the procedure to be successful and for patient morbidity and mortality to be reduced [23]. We compared three distinct thrombus removal methods.

The initial approach involved re-anastomosis after thrombus removal. This method offers a clear view of thrombus color and location during surgery. Using forceps for extraction is more direct and efficient. Furthermore, the anastomosis was re-opened immediately in the case of non-thrombotic closures, such as those caused by suture-related constriction. Anastomotic sutures were partially dissected In the second method, and tirofiban was injected directly into the temporal artery branch and intra-anastomotic space after comprehensive lavage. This approach reduces surgical duration and, compared to direct thrombectomy, results in a lesser degree of injury.

As demonstrated in the literature review, most surgeons chose to have the suture removed at the anastomotic site [2, 12, 15, 20]. Kimura T employed direct surgical embolectomy to rescue 12 patients who developed acute thrombi. Of the 12 patients, 11 had intraoperative reperfusion [12]. Undoubtedly, the outcomes following suture removal at the anastomotic site have been exceptionally positive. However, the first and second approaches necessitate re-opening and re-anastomosing, which presents a challenge to the skills and experience of the surgeon and may expose the patients to the risk of recurrence of thrombosis due to similar endothelial damage or other pathological triggers. The third approach did not disrupt the sutures at the anastomosis. Therefore, compared to the first and second methods, it avoids potential vascular endothelial damage and demonstrates the efficacy of tirofiban in eliminating acute thrombosis. Moreover, this approach minimizes the procedural complexities at the anastomotic site and avoids vascular spasms after repeated manipulations and vessel narrowing following multiple sutures. However, vigilance is necessary for intraoperative and postoperative bleeding complications. In patients subjected to this method, we did not observe bleeding complications associated with tirofiban. Therefore, we consider this method to be both simpler and more efficacious.

### Causes and strategies for anastomotic thrombosis

We categorize the common causes of thrombus formation into two primary types: technical factors and non-technical factors. Technical factors include anastomotic stenosis, back wall suturing issues, endothelial damage, and arterial dissection in donors or recipients. Non-technical factors involve residual blood clots or thrombi resulting from vascular or muscular compression, such as temporal muscle tension or superficial temporal artery folding. If thrombosis is caused by non-technical reasons, the third method is usually effective. In addition to thrombus removal, addressing underlying factors, such as reducing temporal muscle tension and correcting STA folding, is crucial for preventing recurrent thrombus formation. If removing a few stitches can resolve back wall suturing issues or minor vascular dissection, the second method may be employed. If vascular endothelial damage or extensive vascular dissection is identified, the first method is deemed more suitable. Furthermore, to observe if thrombosis recurs, we recommend waiting for 10 min after using each thrombosis treatment method before conducting DSA or ICG imaging.

### Safety and efficacy of Tirofiban infusion

Kang HS et al. used microcatheter-guided selective intra-arterial tirofiban infusion to manage thrombi or emboli occurring during aneurysm procedures. The dose of tirofiban injected into the artery was 0.25–0.50 mg. Among 25 patients with aneurysms, 24 achieved successful revascularization without any associated bleeding complications [10]. Yang et al. employed low-dose intra-arterial tirofiban (0.25-1mg) during Mechanical Thrombectomy (MT) in patients with Acute Ischemic Stroke (AIS), followed by continuous intravenous infusion (0.1ug/kg/min) for 24 h. The occurrence of Symptomatic Intracerebral Hemorrhage (SICH) and Intracerebral Hemorrhage (ICH) exhibited no substantial variances between the tirofiban and non-tirofiban cohorts [24]. Additionally, Junghan et al. reported that tirofiban is not significantly associated with increased rates of intracerebral hemorrhage in patients with acute ischemic stroke [9].

During our surgeries, the dosage of intra-arterial tirofiban administered ranged from 0.15mg to 0.20mg, which was lower than that reported by Kang HS and Yang M. None of the patients who received intra-arterial tirofiban infusion experienced hemorrhagic complications intraoperatively or postoperatively. All patients administered with tirofiban experienced complete dissolution of acute thrombi, with restored arterial patency observed. The final ICG assessment also indicated unobstructed anastomoses. None of the patients exhibited persistent neurological deficits attributed to thromboembolic events.

### Drug selection

Our drug selection is primarily determined by the mechanisms underlying thrombus formation [7]. The recruitment and activation of circulating platelets and the formation of an initial platelet plug (which is frequently observed intraoperatively in white) occur in response to initial tissue injury [2, 6, 7]. Tirofiban is a non-peptide platelet GP IIb/IIIa receptor antagonist, which prevents the aggregation of platelets at atherosclerotic sites by inhibiting the binding of fibrinogen to platelets [18].

Rt-PA, a thrombolytic agent, degrades fibrin, thereby exerting its thrombolytic effect [5], hence its limited efficacy on white thrombosis. Due to the highly destructive nature of intracerebral hemorrhage as a complication of rt-PA therapy [21], we refrained from selecting rt-PA. Takeshi Mikami, Jin Woo Bae, Takahashi JC, et al. have employed heparin intraoperatively to manage acute thrombosis [2, 15, 20]. Additionally, we conducted studies involving the administration of heparin through anastomosis in patients. However, as the thrombus was unresolved, we refrained from using heparin in subsequent studies. Due to the potential risk of intracranial bleeding associated with other medications, the suboptimal effects post-administration of heparin, and the recognized safety and efficacy of tirofiban when applied intracranially, we opted for tirofiban to manage acute thrombosis during STA-MCA procedures.

### Limitations

The small number of patients who have undergone treatment with this technique and the rarity of other studies may lead to an insufficient evaluation of the effectiveness, limitations, and safety of this technique. Higher case series are essential to evaluate this technique further.

## Conclusion

Our method of treatment was efficacious in acute thrombosis management. Intra-arterial administration of tirofiban seems to be a simple and effective treatment option for acute thrombosis during the STA-MCA bypass surgery.

## Data Availability

The raw data supporting the conclusions of this article will be made available by the authors, without undue reservation.
